# Persistence of PFOA Pollution at a PTFE Production Site and Occurrence of Replacement PFASs in English Freshwaters Revealed by Sentinel Species, the Eurasian Otter (*Lutra lutra*)

**DOI:** 10.1021/acs.est.3c09405

**Published:** 2024-05-27

**Authors:** Emily O’Rourke, Sara Losada, Jonathan L. Barber, Graham Scholey, Isobel Bain, M. Glória Pereira, Frank Hailer, Elizabeth A. Chadwick

**Affiliations:** † School of Biosciences, Cardiff University, Museum Avenue, Cardiff CF10 3AX, U.K.; ‡ Centre for Environment, Fisheries and Aquaculture Science (Cefas), Suffolk, Lowestoft NR33 0HT, U.K.; § Environment Agency, Red Kite House, Howbery Park, Wallingford, Oxfordshire OX10 8BD, U.K.; ∥ Lancaster Environment Centre, UK Centre for Ecology and Hydrology, Library Avenue, Bailrigg, Lancaster LA1 4AP, U.K.

**Keywords:** per- and polyfluoroalkyl substances (PFASs), perfluoroalkyl carboxylic acids (PFCAs), perfluoroalkyl sulfonic acids (PFSAs), perfluoroalkane sulfonamides (FASAs), Eurasian otter (Lutra lutra), fluorotelomer sulfonates (FTSs), cyclic PFASs, ether-PFASs, sentinel species

## Abstract

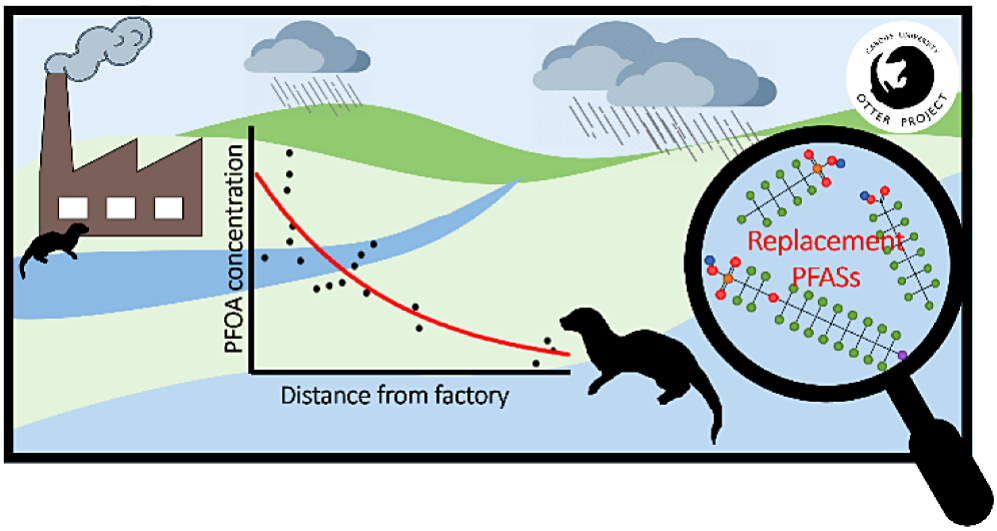

Concentrations of 33 PFASs were determined in 20 Eurasian otters, sampled 2015–2019, along a transect away from a factory, which used PFOA in PTFE manufacture. Despite cessation of usage in 2012, PFOA concentrations remained high near the factory (>298 μg/kg ww <20 km from factory) and declined with increasing distance (<57 μg/kg ww >150 km away). Long-chain legacy PFASs dominated the Σ_33_PFAS profile, particularly PFOS, PFOA, PFDA, and PFNA. Replacement compounds, PFECHS, F-53B, PFBSA, PFBS, PFHpA, and 8:2 FTS, were detected in ≥19 otters, this being the first report of PFBSA and PFECHS in the species. Concentrations of replacement PFASs were generally lower than legacy compounds (max: 70.3 μg/kg ww and 4,640 μg/kg ww, respectively). Our study underscores the utility of otters as sentinels for evaluating mitigation success and highlights the value of continued monitoring to provide insights into the longevity of spatial associations with historic sources. Lower concentrations of replacement, than legacy, PFASs likely reflect their lower bioaccumulation potential, and more recent introduction. Continued PFAS use will inevitably lead to increased environmental and human exposure if not controlled. Further research is needed on fate, toxicity, and bioaccumulation of replacement compounds.

## Abstract 2

Eurasian otters reveal persistence of PFOA pollution close to a fluoropolymer factory which has ceased usage and presence of replacement PFASs in the freshwater environment.

## Introduction

Due to the strength and stability of the perfluorocarbon moiety (C_n_F_2n_), per- and polyfluoroalkyl substances (PFASs) possess unmatched hydrophobic, oleophobic, and temperature-resistant properties. This has led to their widespread use in industrial and commercial applications.^1,2^ Long-chain perfluoroalkyl acids (PFAAs, specifically ≥ C6 perfluoroalkyl sulfonic acids [PFSAs] and ≥ C7 perfluoroalkyl carboxylic acids [PFCAs]) were preferentially selected over short-chain equivalents because of the enhanced properties imparted from multiple C–F bonds.^1^ Extensive use was made of PFOS (a C8 PFSA) in products such as stain repellents and firefighting foam^3^ and PFOA (a C8 PFCA) in fluoropolymer production.^4^ Although advantageous for their uses, the stability of PFASs results in compounds which are not readily degraded in the environment.^1^ The resulting global contamination of PFOS was first demonstrated in 2001,^5^ and since then, numerous studies have shown the ubiquitous presence of PFASs in abiotic and biotic samples in all areas of the world.^6,7^

As knowledge of the persistence, bioaccumulative potential and toxicity of long-chain PFAAs became clear, large manufacturers started phasing out production from the year 2000,^8,9^ and national and international restrictions have been introduced.^10,11^ PFOS, PFOA, and PFHxS have been declared as persistent organic pollutants (POPs) under the United Nations Environment Programme (UNEP) Stockholm Convention in 2009, 2019, and 2022, respectively.^12^ Additionally, C4, C6, and C8 PFSAs and C7–C14 PFCAs are restricted under EU and UK law.^13,14^ Due to the industrially critical applications of PFASs, manufacturers began replacing long-chain PFAAs with alternative perfluorinated compounds.^11,15^ Short-chain (<C6) ether-PFASs (e.g., ADONA, GenX, and EEA-NH_4_) replaced PFOA,^16,17^ and the production of F-53B, which has been used in the electroplating industry in China since the 1970s, increased for use as a PFOS replacement.^18^ Companies have also shifted to using short-chain PFAAs, perfluoroalkane sulfonamides (FASAs), and fluorotelomer sulfonates (FTSs) as replacements.^19−21^ For example, PFBS (a 4C PFSA) and PFBSA (a 4C FASA) have been used to replace PFOS in 3M’s Scotchgard fabric protectors,^19,21^ and 6:2 FTS has been used to replace PFOS in the chrome electroplating industry.^20^ Generally, replacement PFASs have shorter C–F chains and consequently have been marketed as less bioaccumulative and thus safer alternatives,^22^ but these replacement compounds are structurally similar to the substances they replace.^11^

The predominant exposure pathway of PFASs into the environment is via water.^4^ This puts freshwater environments at particular risk, and freshwater wildlife often record the highest concentrations of PFASs compared to terrestrial and marine organisms.^23,24^ Such freshwater contamination also poses a risk to humans, via abstraction of water for drinking and irrigation purposes. Apex predators can provide valuable information on the presence of contaminants in ecological receptors and humans, and they are effective at illustrating large-scale variation in environmental pollution^25^ and thus provide a means of evaluating the success of mitigation measures.^26,27^ In the UK, Eurasian otters (*Lutra lutra*) have been used as a sentinel species for monitoring bioaccumulating chemicals in freshwaters.^28,29^ Otters are apex predators with a predominately piscivorous diet, relatively long lifespan and nonmigratory nature, and the potential to collect samples noninvasively through carcass collection allows for the quantification of spatial and temporal variation.^28,29^ In a previous study, we used the Eurasian otter to examine spatial variation in PFAS concentrations across England and Wales.^29^ PFASs were detected in all otters analyzed, and results suggested wastewater effluent and sewage sludge amended soils are important sources of PFASs to British freshwaters. Concentrations of PFOA showed a highly significant association with a known point source, a factory which until 2012 used PFOA in polytetrafluoroethylene (PTFE) manufacture (AGC Chemicals Europe Ltd., located on the Fylde Coast, Lancashire, UK).^29^ The otters in that study were collected between 2007 and 2009, providing a baseline concentration predating the phasing out of PFOA from usage. To date, little is known about the speed at which PFOA concentrations in aquatic environments might decrease, following cessation of use. This area therefore provided an interesting opportunity to explore the extent to which spatial patterns in pollution, created by past point sources, can have a lasting effect. To test this, we selectively sampled 20 recently (2015–2019) deceased otters from our archive along a transect downwind (east) from the factory location.

Additionally, with such a large number of both legacy and replacement PFASs on the market, most compounds remain un- or underassessed, leaving large data gaps in our understanding of environmental fate and toxicity,^22,30^ and only very few prior wildlife studies have screened for replacement PFASs.^31^ Methodological advances since our previous analysis enabled us to expand the suite of PFASs, which could be quantified, and to determine the concentrations of some replacement PFASs in the environment around the factory, as well as PFOA and other legacy PFASs. We hypothesized that (1) due to the extreme persistence of PFOA, the association with the factory would still be detectable, and (2) replacement PFASs would be present in the otter tissues, in addition to the legacy PFASs previously identified.

## Materials and Methods

### Otter Sample Selection

**Figure 1:**
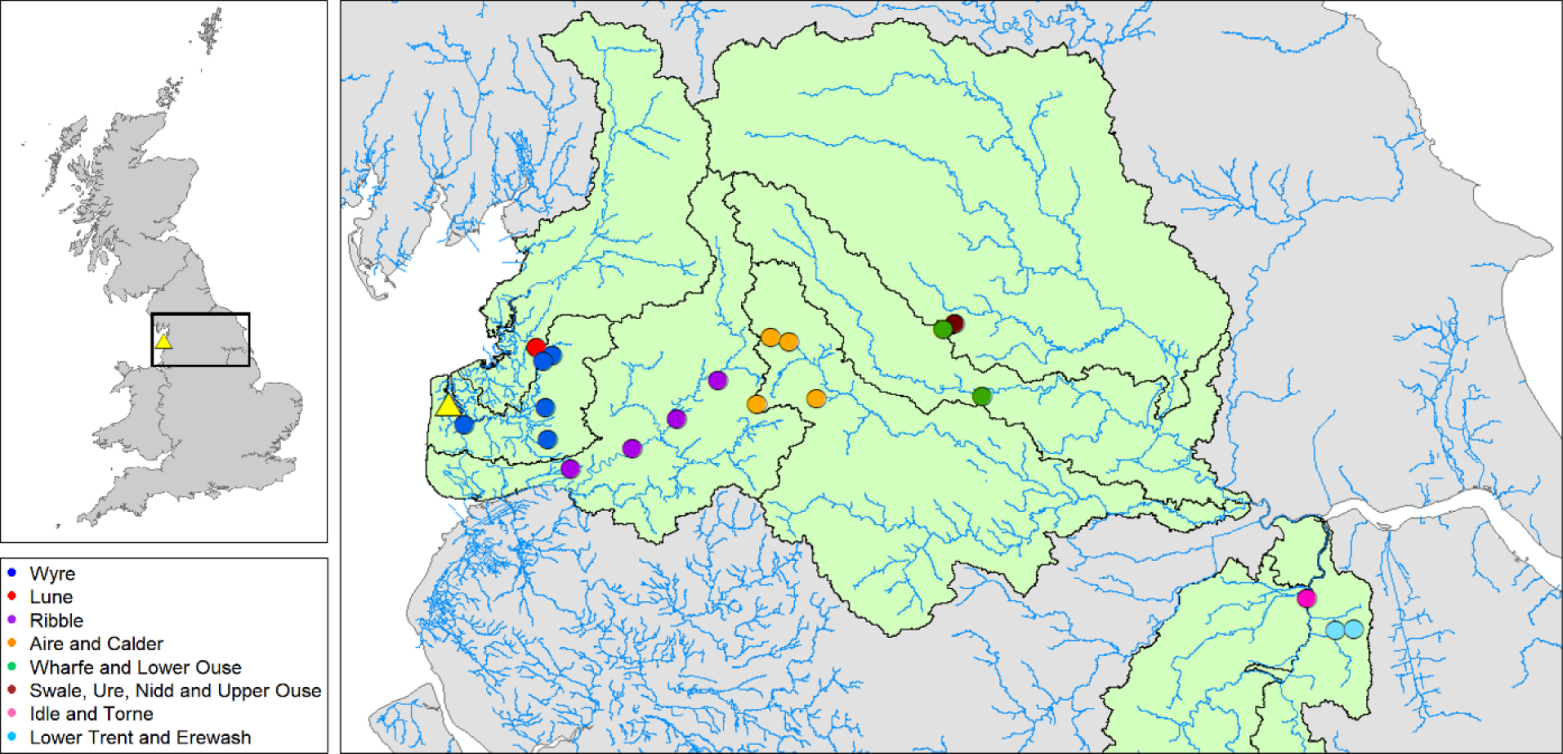
Location of factory (yellow triangle) and otters selected for analysis (circles). Otters are color-coded by the river catchment they were found in. River catchments with otters selected for this study are highlighted in green with gray boundaries. Rivers are shown as thin blue lines. The factory location of AGC Chemicals Europe, Ltd., is indicated by the yellow triangle. Map produced using ArcMap 10.7.1.

Otters found dead were collected as part of the Cardiff University Otter Project. At collection, each otter’s location (National Grid Reference) and date found were recorded. During a standardized postmortem examination, biometric data (including sex, age-class, length, weight, and reproductive status) and tissue samples were collected. Samples were archived in individual grip seal bags at −20 °C. Liver was selected as the optimum tissue for comparability with previous studies,^23,24,32^ and because detection in liver has been shown to be higher than other tissues and therefore more likely to be of a detectable concentration.^32^ Otter selection was restricted to a transect running east away from the PTFE manufacturing facility on the Fylde coast, North England. A sample of 20 livers from otters which died between 2015 and 2019 were selected for analysis (Figure 1). This selection excluded juvenile otters and those with gross evidence of decay as determined at postmortem. More details of sample selection are available in Table S1.

### Analytical Determination

Frozen liver subsamples were sent to the Centre for Environment, Fisheries and Aquaculture Science (Cefas), Lowestoft, UK, for analysis of a suite of 33 PFAS compounds. Analyses were performed on individual otter livers using an ultraperformance liquid chromatograph Acquity (Waters Ltd., Elstree, Hertfordshire, UK) with an isolator column XBridge C18 (50 mm × 2.1 mm and 3.5 μm particle size), separation was achieved using a BEH C18 analytical column (50 mm × 2.1 mm and 1.7 μm particle size). The UPLC system was coupled to a TQ MS Xevo triple quadrupole mass spectrometer (Waters Ltd.), using an electro spray ionization (ESI) probe in negative mode. Details of extraction and cleanup were as reported previously^29^ with additional internal standards to facilitate the increase from 15 target compounds quantified previously, to 33 quantified here, of which 15 are identified as “replacements” (target compounds, analytical standards, and limits of quantification (LOQs) are listed in full in Table S2). As previously, for quality assurance purposes, a blank and reference material sample were analyzed with every 10 samples; the current analysis used NIST 1946 (Lake Superior fish tissue) and NMCAG-RM1 spiked mussel tissue, where the previous study used spiked flounder. For PFOS and PFHxS, Cefas have standards for both linear and branched PFHxS and PFOS, and therefore, both isomers are reported. For other isomeric determinands, only the linear isomer was quantified and reported. Further details of analytical determination are provided in the Supporting Information.

### Data Analysis

For the purposes of data analysis, samples below the limit of quantification (LOQ, see Table S2) were assigned 0.5xLOQ. All statistical analyses were carried out in R (version 4.1.2). To explore the association of PFOA concentration with distance from the PTFE manufacturing site, it was important to control for confounding variables. The ad hoc nature of sample collection of an internationally protected species brings some limitations, i.e., constraining sample size and preventing an even distribution of samples along a geographically linear transect. Temporal, spatial, and biotic variation between individuals are potentially confounding variables, and to address this, we used multivariate analysis using generalized linear modeling (GLM). Sample size precluded simultaneous inclusion of all potential confounding variables that we considered plausible. However, our previous publication,^29^ which had a larger sample size and more widespread spatial distribution of samples, explored additional potentially confounding biotic variables (sex, length, and body condition) and showed no effect. In the present study, we therefore chose to omit these variables, after running preliminary checks to confirm that there were no significant associations, determined by a model of PFOA concentration with sex, length, and body condition.

We modeled PFOA concentration as the dependent term, with distance from PTFE manufacturing site as an independent term in a GLM. We included year of otter death as a continuous variable (to control for potential change between 2015 and 2019), latitude of otter death (to control for north–south variation away from the transect), and wastewater treatment works (WWTW) load (to control for an alternative potential source, see Table S3 for further details). Model validation was carried out to check assumptions of normality, homoscedasticity, and leverage; raw data with Gamma error family and log link function resulted in the best model fit and are reported. One otter with a high PFOA concentration (1000 μg/kg ww) and WWTW load value (134,791.3 PE) caused excessive leverage to the model (based on Cook’s distance estimate); we ran the model with and without this otter and compared the results; the nature of the association with the PTFE manufacturing site did not change (Table S5, Figure S3). Results reported are those excluding this individual. As can be seen in Figure 1, three otters in the sample are much further south than the other otters. This is due to the ad hoc nature of otter carcass collection (no otters further north were available for selection). To ensure the southern latitude of these otters did not influence the result, we included latitude in our models and also modeled the data both with and without these otters for comparison. Removal of these otters did not alter the nature of the association with the PTFE factory, and results presented include those southern individuals (Table S5, Figure S4).

Determination of the most important variables in the models was achieved using multimodel inference; independent variables were standardized using the standardize function in the Arm package;^33^ the dredge function in the MuMIn package^34^ was then used to rank models by AICc, and model averaging was applied to models where delta AICc was <2.^35^ The full average method, whereby parameter averages are calculated using the total number of top models and setting the parameter to zero in models it does not appear in, was used to determine model estimates.^36^ We reported all independent variables retained in the top models and their relative variable importance, as well as significance (*p* values) in Table S5. In deriving model predictions, we did not remove variables based on *p* values <0.05 but instead used the full starting model using the “predict” function in R, with latitude, WWTW load, and year controlled to their mean values.

We also modeled other PFASs in order to evaluate whether there was a general west to east decline of all PFASs (as opposed to a specific association of PFOA with factory location), or whether we could detect any trend in other PFASs which might also be associated with PTFE production at the site. We carried out a principal component analysis (PCA) to explore correlations between contaminants which were detected in ≥70% of otters. Data were log transformed prior to analysis to avoid undue influence of outliers. The singular value decomposition method (prcomp) was used for calculating the components, and contaminant concentrations were scaled (to means of zero and variance of one) to avoid emphasis on chemicals with greater variance. Results of the PCA showed that most PFASs, except for PFBA, PFOA, and PFNA, loaded substantially on PC1, which accounted for 53.5% of the variation (Figure S1). We used PC1, PFNA, and PFBA as dependent variables in additional GLMs with the same model design as described above for PFOA (PFNA and PFBA were modeled individually because these PFCAs were not well represented by PC1).

## Results and Discussion

### Association of PFOA with PTFE Manufacturing Facility

**Figure 2:**
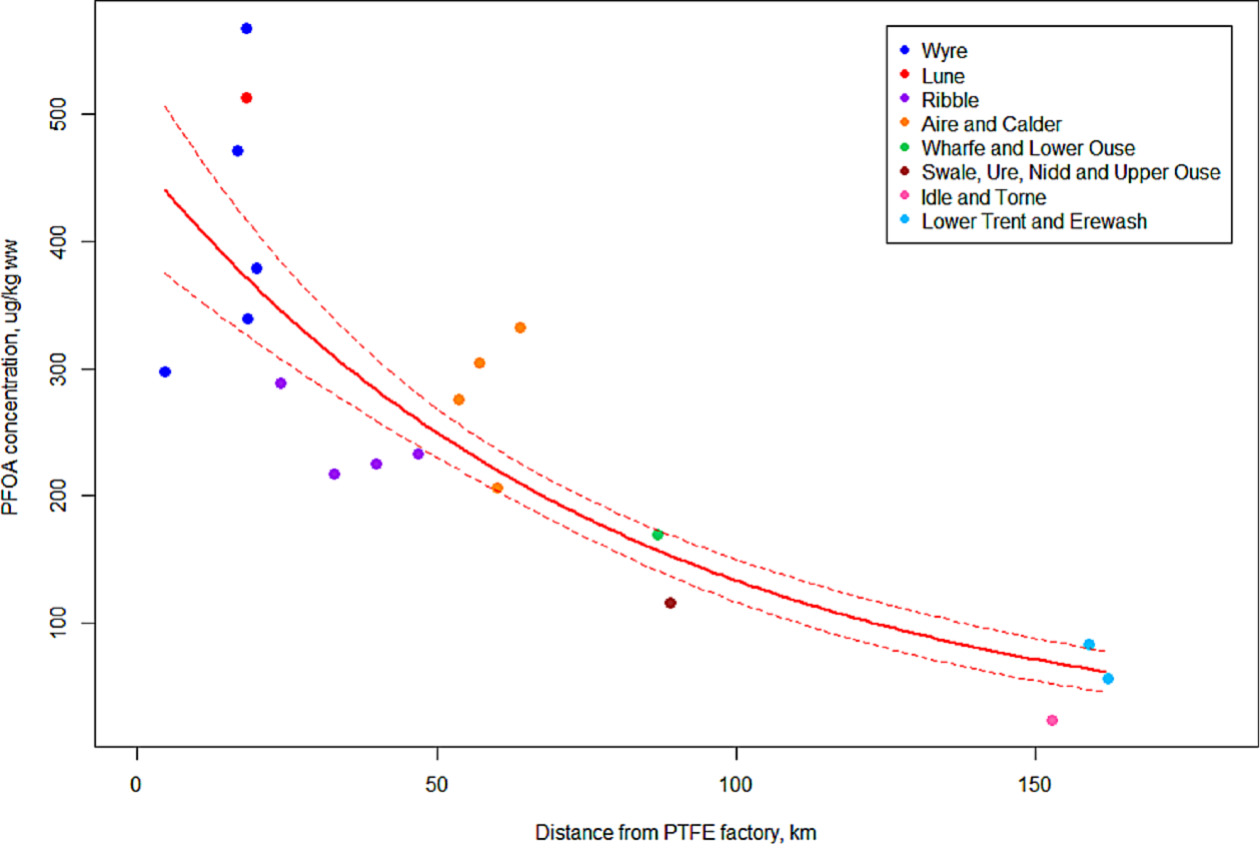
Model-predicted decline in PFOA concentration with distance from the factory producing PTFE. Colored dots show the raw data, color-coded by river catchment. Solid red line shows the model predicted concentrations and dotted lines show standard error. One leverage point has been removed for modeling (*n* = 19), and year, latitude, and WWTW load are controlled to their mean values, see statistical methods for details.

Despite cessation of usage of PFOA by the PTFE manufacturing factory in 2012,^37^ PFOA concentrations in the otters found dead between 2015 and 2019 still showed a pattern of significant decline with distance from the fluoropolymer factory (averaged model *n* = 19: relative variable importance [RVI] = 1, z = 8.449, *p* = < 0.001, Table S5). High concentrations of between 298 and 568 μg/kg ww were seen within 20 km of the factory, and the lowest concentrations of <57 μg/kg ww were seen in otters >150 km away from the factory (Figure 2). The highest concentrations seen in this study were higher than the maximum value seen in our previous study which was 130 μg/kg ww in an otter found in 2007, 46 km from the factory.^29^ We assume this to reflect the locations of the otters, rather than reflecting a temporal increase; samples for the current study were mostly located closer to, and downwind of, the factory, whereas the previous study sampled otters from across England and Wales (Figure S2). The gradual decline in concentration with distance from the factory in otters from across eight different river catchments (Figure 2) further supports our previous conclusion (in O’Rourke et al. 2022) that air dispersal with the prevailing eastward wind direction was an important pathway for PFOA contamination. Effluent from the factory is released into the tidal waters of the River Wyre estuary. Therefore, if effluent was the sole source of contamination, we would expect a much sharper decline in concentration in the otters, with high concentrations in otters with a home range covering the estuary area, and much lower concentrations in otters with home ranges that do not, rather than a gradual decline eastward. Other PFASs (PC1, PFBA, and PFNA) showed no association with distance from the factory (distance to factory was retained for PC1 but had a very low relative variable importance [0.09], distance was not retained for either PFBA or PFNA, Table S5). This supports our interpretation that the factory was a point source of PFOA, rather than there being a general west to east decline of all PFASs. While we cannot rule out a coastal effect contributing to the observed gradient, e.g., from an impact of sea spray aerosol,^38^ a gradient should also be seen for other PFASs if this was an important driver in this area.

The clear spatial association is expected to remain for many years due to PFOA’s persistence in the environment.^1^ Evidence suggests PFOA will gradually migrate through soil to groundwater^39^ and thus become less bioavailable; biomonitoring of otters in the area will provide a valuable case study to determine how long associations with industrial point sources remain after cessation of use. The variable “Year” was retained, with a negative (although nonsignificant, *p* > 0.05) association, in one of the two top models for PFOA (RVI: 0.37, Table S5). This may suggest the start of a decline in environmental concentrations, but the relatively small sample size and short time span (5 years) in our study provide very limited power to detect temporal change (which was not the aim of this research), and further research is warranted to explore change over time. Year was also retained (with a negative but nonsignificant association) in models of PFNA and the other PFASs (represented by PC1, of which the majority are legacy PFASs), whereas it was not retained in the model for PFBA, a replacement PFAS (Table S5).

Comparing previous data and model predictions^29^ with those from the current study (Figure S2) suggests that at distances >100 km from the factory, predicted concentrations in more recent years are slightly lower than those from 2007 to 2009. Model predicted concentrations <100 km from the factory differed significantly between the two studies, emphasizing the importance of monitoring PFOA concentrations close to point sources in order to capture the highest concentrations. PFOA is less mobile in water^10^ and has a lower potential for long-range atmospheric transport^40^ than shorter chain PFASs, and thus, the highest concentrations are found in the immediate vicinity of a source.^39^ Sampling on a transect with wind or water direction is also important to identify areas of highest exposure; the two samples closest to the factory in the previous data set had much lower concentrations than those at similar distance in the current study but were much further north of the factory (not downwind).

During model validation steps, we found that in this sample, the percentage of arable land was positively correlated with distance from the factory and therefore removed this variable from our starting model due to collinearity (see Table S3). Although we were therefore unable to explicitly test (or control) for an association with arable land, our previous modeling (covering a broader area, and with larger sample size) suggested that arable land had a positive (but not significant) association with PFOA.^29^ In the current study area, increasing arable land cover would therefore be expected to drive an increase in PFOA with distance from the factory. Instead, we see the reverse; therefore, we are confident that high PFOA concentrations reflect the factory point source and not agricultural inputs. In our previous study, a significant positive association was also found between PFOA and wastewater treatment works load (WWTW); this variable was not retained in the top models for PFOA (*n* = 19) in the current study. Latitude was also not retained in the top models (*n* = 19) for PFOA (for full model outputs see Table S5).

The AGC Chemicals Europe Ltd. factory stopped using PFOA in 2012, and now use ammonium difluoro[1,1,2,2-tetrafluoro-2-(pentafluoroethoxy)ethoxy]acetate, an ether-PFAS known as EEA-NH_4_. The company is permitted to import EEA-NH_4_ from Japan at volumes of up to 100 tonnes/year for use as a processing aid in PTFE manufacture.^41^ There are no other REACH Registrants in the UK or EU for EEA-NH_4_.^41^ Wastewater from the factory drains into an effluent pit before being discharged into the River Wyre (which flows west to the coast), and waste gases are passed through an aqueous scrubber with an efficiency of removal of between 41.1 and 99.97%; therefore, contamination of surrounding water and air is possible.^41^ Analysis of EEA-NH_4_ was not conducted in this study due to the unavailability of the analytical standard at the time of analysis. The Environment Agency analyzed 46 marine fish samples in 2022 from coastal sites near the factory and did not find detectable concentrations of EEA-NH_4_ (LOD: 0.12 μg/kg, unpublished data supplied by the Environment Agency). However, PFOA is also undetected in fish samples from across England (data from 78 sites, collected 2014–2019, LOD: 1 μg/kg)^42^ despite being ubiquitous in otter samples (100% of 50 samples collected 2007–2009 across England and Wales^29^ and 100% of samples in the current study); therefore, the absence of evidence for EEA-NH_4_ in fish samples is not evidence for absence in the environment. We do not know of any studies which have examined EEA-NH_4_ in apex predators. EEA-NH_4_ has similar physicochemical properties to ADONA and GenX^43^ which are used as processing aids by 3 M and Chemours, respectively. Both have been detected around the factories where they are used.^15,44,45^ An important next step would be to include EEA-NH_4_ in analysis, so we can understand presence and bioaccumulation of this replacement compound.

### The Chemical Mixture of Legacy and Replacement PFASs

Thirty-three PFASs were quantified (including linear and branched isomers of PFHxS and PFOS). We have divided these compounds into two groups: *legacy PFASs*, which have been in production for many years and most of which are now restricted, and *replacement PFASs*, which have increased in production to replace legacy compounds. The groupings can be seen in Table 1.

**Table 1:** Descriptive Statistics for Each of the PFASs Analysed^a^

compound	C_n_	class	*n* > LOQ	min	max	median	mean
legacy PFASs							
L-PFOS	8	PFSA	20	645	4640	1740	1700
PFOA	8	PFCA	20	24.1	1000	283	305
PFDA	10	PFCA	20	91.7	573	213	228
PFNA	9	PFCA	20	71.7	760	196	217
B-PFOS	8	PFSA	20	40.7	468	182	177
PFUnA	11	PFCA	20	27.4	156	45	53.4
PFDoDA	12	PFCA	20	8.86	149	28.4	34.6
PFHpS	7	PFSA	20	6.04	36.8	17.1	17.9
PFOSA	8	FASA	20	3.32	412	15.5	39.0
L-PFHxS	6	PFSA	20	3.38	62.5	12.6	17.6
PFTrDA	13	PFCA	20	3.79	27.6	6.69	7.90
PFTeDA	14	PFCA	20	1.65	40.6	5.17	7.42
PFDS	10	PFSA	20	0.344	136	2.23	9.60
PFNS	9	PFSA	20	0.048	31.1	1.66	3.14
PFHxSA	6	FASA	14	<LOQ	30.7	0.547	2.11
B-PFHxS	6	PFSA	20	0.0458	2.47	0.218	0.505
N-EtFOSAA	8	FASAA	2	<LOQ	0.15	0.05	0.0753
N-MeFOSAA	8	FASAA	5	<LOQ	0.108	0.025	0.0393
replacement PFASs							
PFBSA	4	FASA	20	0.443	17.1	2.32	3.42
PFECHS	8	cyclic-PFAS	20	0.226	4.42	0.949	1.35
8:2FTS	10	FTS	19	<LOQ	70.3	0.817	4.52
PFBS	4	PFSA	20	0.1	2.09	0.373	0.585
PFHpA	7	PFCA	19	<LOQ	4.24	0.364	0.655
6:2 Cl-PFESA (F-53B major)	8	ether-PFAS	19	<LOQ	1.33	0.33	0.467
PFPeS	5	PFSA	17	<LOQ	2.1	0.25	0.545
PFBA	4	PFCA	16	<LOQ	0.644	0.206	0.237
6:2FTS	8	FTS	2	<LOQ	5.99	0.05	0.0582
PFHxA	6	PFCA	3	<LOQ	0.344	0.05	0.503
8:2 Cl-PFESA (F-53B minor)	10	ether-PFAS	6	<LOQ	0.134	0.025	0.0340
PFPeA	5	PFCA	2	<LOQ	0.158	0.025	0.0459
HPFO–DA (GenX)	6	ether-PFAS	0	<LOQ	<LOQ		
NaDONA (ADONA)	6	ether-PFAS	0	<LOQ	<LOQ		
4:2FTS	6	FTS	0	<LOQ	<LOQ		

a Concentrations are recorded in μg/kg wet weight (ww). Compounds and classes are denoted by their abbreviation, see Table S2 for full compound and class names. Compounds are grouped into legacy and replacement PFASs and ordered by decreasing median concentration within those groups. Mean concentrations are also provided for comparison with other studies but are less representative of the highly skewed data distributions.

**Figure 3:**
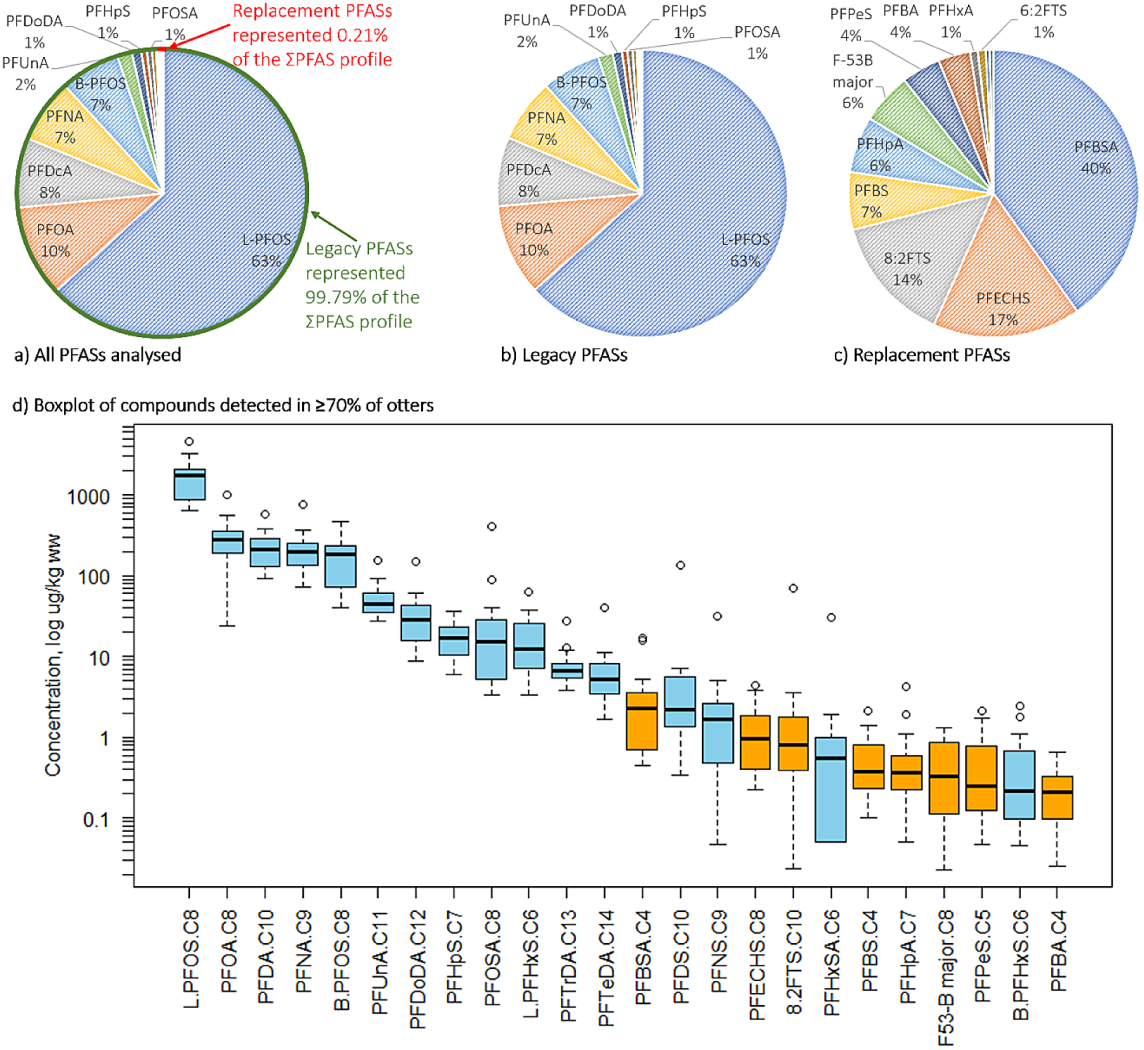
Proportion of individual substances in relation to the total concentration of a) all PFASs, b) legacy PFASs, and c) replacement PFASs. Compounds which represented <1% of the profile are not labeled. d) Concentrations of compounds with detection frequency of 70% and above. Compounds are denoted by their abbreviation and carbon chain length, see Table S2 for full compound names. Concentrations are recorded in μg/kg ww, plotted on a log scale. Blue = legacy compounds, orange = replacement compounds, presented in order of decreasing median concentration. Concentrations are presented as a boxplot; the thick black line indicates the median concentration, the lower and upper extents of the box indicate the 25th (Q1) and 75th (Q3) percentiles of the data distribution, whiskers show the lowest and highest values excluding outliers, and circles indicate outliers (1.5× the interquartile range).

Of the 18 legacy PFASs analyzed, 15 were detected in all 20 otters, these were the 7 PFSAs, 7 PFCAs, and PFOSA. PFHxSA, NMeFOSAA, and NEtFOSAA were detected in 14, 5, and 2 otters, respectively. Of the 15 replacement PFASs analyzed, PFBSA, PFECHS, and PFBS were detected in all 20 otters; PFHpA, F-53B major, and 8:2 FTS in 19 otters; PFPeS and PFBA in 17 and 16 otters, respectively; and the other 7 compounds (F-53B minor, PFPeA, PFHxA, 6:2 FTS, 4:2 FTS, Gen-X, and ADONA) in fewer than 6 otters (Table 1). Generally, the legacy compounds had much higher concentrations than the replacement compounds (Figure 3d). Linear PFOS (L-PFOS) had the highest median concentration of 1740 μg/kg ww, whereas replacement compounds all had median concentrations of <2.4 μg/kg ww (Table 1). These differences are likely to reflect both their chemical properties (it is suggested that replacement PFASs are less bioaccumulative)^17^ and the shorter period of time over which they have been used.

Associations between PFASs with a detection frequency ≥70% were examined by principal component analysis (PCA). Most of the long-chain PFAAs loaded most heavily (negatively) on PC1 and short-chain PFAAs and PFBSA loaded more heavily (positively) on PC2 than PC1 (Table S4). This difference between carbon chain length may reflect differing retention times in biota (short-chain compounds are less bioaccumulative and are eliminated more quickly from the body).^10,17^ The 8-carbon replacement compounds PFECHS and F-53B major both loaded heavily on PC1 along with legacy long-chain PFSAs and PFCAs, including PFOS. In addition to having similar retention times in biota as the long-chain legacy compounds, this could reflect the use of PFECHS and F-53B as replacements for PFOS and consequently their similar environmental pathways into freshwaters. In our previous study, PFOS concentration was significantly positively correlated with wastewater treatment works load and arable land suggesting wastewater effluent and sewage sludge amended soils are important sources of PFOS to the environment.^29^ Studies have shown that, like other PFASs, F-53B is not successfully removed at wastewater treatment plants and is consequently present in effluent^46^ and sewage sludge.^47^

### Continued Dominance of Legacy PFASs, Especially PFOS

Despite recent phase outs and regulation, long-chain PFSAs and PFCAs were detected in all otters analyzed and are still seen at the highest concentrations. The top five PFASs (highest median concentrations) were L-PFOS > PFOA > PFDA > PFNA > B-PFOS (Figure 3d); these compounds had median concentrations of >181 μg/kg ww, and all other compounds had median concentrations of <45 μg/kg ww (Table 1). These five compounds are all regulated under the Stockholm Convention and/or UK/EU REACH.^12−14^

L-PFOS dominated the ΣPFAS profile; 63% of all PFASs analyzed was L-PFOS, and B-PFOS also represented a high proportion of the profile (7%, Figure 3a). PFSAs are more bioaccumulative than PFCAs of the same fluorinated carbon chain length^48^ which explains why PFOS (PFSA, 8 fluorinated carbons) dominates over PFNA (PFCA, 8 fluorinated carbons) and the shorter fluorinated carbon chain PFOA (PFCA, 7 fluorinated carbons) despite all being extensively used. PFOS was restricted under the Stockholm Convention in 2009^12^ but is still commonly seen at the highest concentrations in otters and other apex predators,^23,24^ making PFOS one of the predominant organic contaminants in the environment.^49^ The median concentration of L-PFOS seen in the otters was 1740 μg/kg ww; this is similar to the result seen in otters from the UK found dead in 2016 and 2017 (1340 μg/kg ww, n = 5).^23^ It is higher than the median seen from 50 otters found dead between 2007 and 2009 (820 μg/kg ww),^29^ but this is likely due to a more rurally distributed sample in the previous study compared with the current selection, rather than a temporal increase. Some biotic studies have demonstrated a decline in PFOS since regulation, while others show no trend.^50^ A systematic review of biotic data concluded that overall PFOS concentrations do not yet appear to be declining on a global scale.^50^ The half-life of PFOS in the environment may exceed 41 years,^51^ so it could take many years until significant declines in the environment are seen.

Long-chain PFSAs and PFCAs are extremely persistent, and therefore, extensive historic use and emissions has led to high concentrations in the environment.^15^ In addition, PFSAs and PFCAs are terminal degradation products, and concentrations in the environment are increased by the degradation of precursor PFASs.^1^ It has been estimated that worldwide emissions of PFOS to the environment were 450–2,700 tonnes between 1970 and 2002, compared to 6,800–45,250 tonnes for PFOS precursors over the same period.^3^ PFOSA (a C8 FASA) was an ingredient in 3M’s Scotchguard formulation from 1956 to 2003,^52^ and it degrades to PFOS in the environment. Degradation rates vary between species and are more rapid in carnivores than fish.^53^ The predominantly piscivorous diet of otters^54^ makes PFOSA in fish an important exposure route to PFOS for otters. In our study, PFOSA was detected in all the otters analyzed and had the eighth highest median concentration (15.5 μg/kg ww) of the PFASs analyzed (Figure 3d). PFHxSA (a C6 FASA) has been detected in some firefighting foams along with PFOS,^55^ and it is also a precursor of PFHxS. PFHxSA is not often included in biota studies but was detected in 14/20 otters in our study at concentrations up to 30.7 μg/kg ww. PFHxS is listed under Annex A of the Stockholm Convention,^12^ and therefore, the detection of PFHxSA in the otters of our study suggests the compound could be an important exposure pathway for PFHxS in ecological receptors. N-MeFOSAA and N-EtFOSAA are transformation products which degrade to PFOS;^49^ they were rarely detected in the analyzed otters (5 and 2 otters respectively) and only seen at very low concentrations.

### Emergence of Replacement PFASs

The rank order by median concentrations of the replacement PFASs analyzed in this study and detected at ≥70% is PFBSA > PFECHS > 8:2 FTS > PFBS > PFHpA > F-53B major > PFPeS > PFBA (Figure 3d). Concentrations of the replacements were generally lower than those of the legacy compounds (median range replacements: < LOQ-2.315 μg/kg ww, median range legacy: < LOQ-1740 μg/kg ww). The sum of replacement compounds only accounted for 0.21% of the Σ_33_PFAS profile (Figure 3a); however, their presence clearly demonstrates their bioavailability to apex predators, and continued use will inevitably lead to increased environmental and human exposure if not controlled. Despite their relatively low concentrations, we therefore provide a brief overview of each.

PFBSA was seen at the highest concentrations, accounting for 40% of the profile for replacement compounds (Figure 3c). PFBSA (a 4C FASA) is a likely precursor to PFBS^21^ which is listed as a substance of very high concern (SVHC) under UK REACH.^14^ PFBSA and PFBS were both detected in all otters in this study, but PFBSA was seen at higher concentrations. This difference in concentrations may be due to greater metabolism of PFBS in otters, and therefore, PFBS (acquired directly or via metabolism of precursors) is eliminated more quickly than PFBSA.^21^ There is very limited understanding of its presence in, and toxicity to, biota; to our knowledge, this is the first report of PFBSA in Eurasian otters.

PFECHS was detected in all liver samples, and it accounted for 17% of the replacement PFAS profile (Figure 3c) with a median concentration of 0.949 μg/kg ww (Figure 3d). To our knowledge, this is the first report of PFECHS in Eurasian otters. A previous study on mammals from Norway did not find detectable concentrations of PFECHS in any of the Eurasian otter liver samples, nor the American mink, wolf, moose, roe deer, or Arctic fox liver samples also analyzed in the study, but did detect it in polar bear blood serum samples with concentrations ranging from 0.26–3.09 ng/mL.^24^ PFECHS has been detected in polar bear liver^56^ and in ringed and harbor seal liver,^57^ but no quantitative data were stated by the studies. PFECHS has also been recorded in human blood^58^ fish and abiotic samples.^59^ PFECHS is an 8-carbon cyclic PFAS and is considered an analogue of PFOS due to their similarities in structure and properties.^22^ PFECHS has no known precursors, so presence in the environment must be through its use.^60^ Commercially, it has been used to replace PFOS as an erosion inhibitor in aircraft hydraulic fluids^61^ and has been detected at the highest concentrations around airports where PFECHS-containing fluids are heavily used^59,62^ but has also been detected in the Arctic, where there is no local source, suggesting PFECHS can undergo long-range oceanic transport.^60^ As with other replacements, PFECHS has been marketed as less accumulative and thus safer, but an *in vitro* study on acute toxicity in fish cells showed that while little PFECHS was concentrated into cells, it did cause adverse effects,^63^ suggesting that reduced bioconcentrative and bioaccumulative potential does not always correspond to a reduction in toxicity.^63,64^

Three fluorotelomer sulfonates (FTSs) were analyzed in this study; 8:2 FTS was detected in 19/20 otters, 6:2 FTS in 2 otters, and 4:2 FTS was not detected. These are similar to detection frequencies to those found in the liver of American river otters (*Lontra canadensis*, 79%, 12%, and 0%, respectively).^32^ 8:2 FTS had the third highest median concentration and the highest maximum concentration of the replacement compounds (median: 0.817 μg/kg ww, maximum: 70.3 μg/kg ww, Figure 3d); this is higher concentrations seen in Eurasian otters from Europe (maximum: 14.5 μg/kg ww)^23^ and American river otters (maximum: 1.87 μg/kg ww).^32^ FTSs have been used as PFOS replacements in a wide variety of products^17,20,65^ but are probably excreted quickly,^32^ and FTSs degrade to PFCAs in the environment and biota.^66,67^ The rate of degradation increases with decreasing fluoroalkyl chain length under ultrasound thermolysis experiments,^68^ and this may explain the higher detection frequency of 8:2 FTS compared with 6:2 and 4:2 FTS.

Of the short-chain PFAAs, PFBS (C4), PFPeS (C5), PFBA (C4), and PFHpA (C7) were all detected in ≥16 otters; PFPeA (C5) and PFHxA (C6) were detected in 2 and 3 otters, respectively (Table 1). Concentrations in this study were lower than those of long-chain PFAAs (range long-chain: 0.046–4640 μg/kg ww, range short-chain: < LOQ-4.24 μg/kg ww), which aligns with our previous study^29^ and with other wildlife studies.^23,32,69^ This likely reflects both the lower bioaccumulation potential^11,70^ and more recent increase in use of short-chain PFASs. Implementation of regulation is beginning in Europe for some of the short-chain PFAAs, with PFBS and PFHpA now added to the EU REACH Candidate List of substances of very high concern (SVHC) since January 2020 and January 2023, respectively, and PFHxA is proposed for addition.^13^ As terminal degradation products of other PFASs, however, precursors degrade to PFCAs and PFSAs and thus add to the environmental burden.^11^ Studies have demonstrated that short-chain PFASs are as persistent as long-chain.^11,71^ They are also more mobile than long-chain compounds and sorb less to sediments; therefore, they are more bioavailable to wildlife^10,11,72^ and will likely be more of a contamination risk to drinking water supplies.^40,73^ Additionally, the compounds themselves and their volatile precursors may undergo long-range atmospheric and oceanic transport, thus posing a greater risk to remote areas.^24^ Studies have shown short-chain compounds to be less toxic than long-chain PFAAs.^11,30^

Four fluoroalkylethers (ether-PFASs) were included in this study, these were 6:2 Cl-PFESA (F-53B major), 8:2 Cl-PFESA (F-53B minor), HPFO–DA (Gen-X), and NaDONA (ADONA). F-53B is formed of two components: F-53B major and F-53B minor; in our study, F-53B major was detected in 19 of the 20 otters with concentrations up to 1.33 μg/kg ww, and F-53B minor was detected in 6 otters with concentrations up to 0.13 μg/kg ww. F-53B major has been previously reported in surface water and sediment samples in the UK^74,75^ and in one otter from the UK at a concentration of 3.3 μg/kg ww^23^ and in American otter liver at a maximum concentration of 0.06 μg/kg ww.^32^ F-53B is not manufactured in the UK or EU, usage of the chemical is not known, and it is not registered under the EU or UK REACH Regulations, so no single company can be importing it in quantities exceeding 1 tonne/year.^76^ Therefore, F-53B is likely mainly entering European environments via the use and disposal of imported products containing F-53B, the low volume importation of the chemical for use in the chrome plating industry,^76^ and/or via long-range oceanic transportation from China.^2^ Although long-range transport potential of F-53B is considered low (it has been calculated that 0.02–0.50% of annual F-53B emissions reach the Arctic via oceanic advection),^2^ it has been detected in mammals from Greenland where there is no direct source.^77^ As F-53B production increases in China in response to the cessation of PFOS, global concentrations in remote locations and countries where it is not produced will likely increase. Originally, F-53B was marketed as less persistent, bioaccumulative, and toxic compared to PFOS. However, data suggest that F-53B likely meets the very persistent criteria of the REACH Regulation^76^ and has been reported as the most biopersistent PFAS in humans to date, with a half-life of 15.3 years, compared to 3.4 years for PFOS.^18^ In addition, the bioaccumulation potential may be at least that of PFOS, if not greater^31^ with a study showing the mean log bioaccumulation factors of F-53B and PFOS to be the same in Crucian carp.^62^ F-53B is suggested to have similar acute toxicity as PFOS and is considered harmful to aquatic life.^46^ In China, where F-53B use has increased, studies on humans have shown blood concentrations to be increasing.^18^ In Europe, the PFOS replacement PFHxA is currently subject to an EU restriction proposal, and thus, the English Environment Agency warns that companies could shift to alternatives like F-53B, which would increase use and emissions.^76^ Neither ADONA nor GenX was detected in any of the otters in this study. They were formulated by 3 M and DuPont (now Chemours), respectively, in response to the cessation of PFOA, to be used as processing aids in the manufacture of fluoropolymers. Neither 3 M nor Chemours manufactures fluoropolymers in the UK. GenX has been mainly detected in surface water and plant samples from around the factories where they are used^44,45^ and has also been detected in river water in the UK,^74^ suggesting exposure from the use and disposal of GenX-containing products. ADONA is generally not detected in surface water samples^45,74^ but has been found close to a factory in Germany.^15^ There are very limited wildlife studies including ADONA and GenX; ADONA has been detected in deer liver in Germany,^15^ and GenX has been detected in common carp (*Cyprinus carpio*) in China^78^ and striped bass (*Morone saxatilis*) in USA;^79^ in other studies, they have been below limits of detection.^23,31^ ADONA and GenX are thought to be less bioaccumulative than PFOA;^31^ however, they have been on the market a much shorter time than PFOA, and concentrations currently below limit of detection may reach detectable levels as emissions continue.^15^ Limited toxicological data from rat studies has suggested that while ADONA may be less toxic than PFOA^80^ GenX has been shown to be more toxic, potentially being as toxic as PFOS.^64,81^

### Implications for Further Research

Our research illustrates the value of Eurasian otters as sentinels of bioaccumulating contaminants in the freshwater environment. Importantly, we document the continuing dominance of legacy PFASs, despite regulation, and the presence of replacement compounds. Further biomonitoring of PFOA in proximity to the factory is warranted, to determine how long associations with industrial point sources remain after cessation of use. Additionally, concentrations of EEA-NH_4_ should be quantified to determine the presence, and possible association with the factory, of this PFOA replacement.

Further research is needed to explore change over time of PFASs, to understand the impact of regulation on legacy compounds, and the introduction of replacements. In our study the replacement PFASs were all seen at relatively low concentrations, but PFBSA, PFECHS, PFBS, 8:2 FTS, PFHpA, and F-53B major were all detected in ≥19 of the 20 otters sampled. Despite a relatively small sample size and study area, this ubiquitous presence of PFASs in the otters is concerning. The detection of substances that are not produced or used in manufacturing processes in England demonstrates the global issue of PFASs. Growing evidence of the presence of replacement PFASs in remote locations away from production and human population, e.g., the Arctic, suggests many replacements are, or have the potential to become, globally ubiquitous contaminants.^15^ The increased production, use, and emissions of replacements will inevitably lead to increased environmental and human exposure. It will therefore take many years for global environmental levels to respond to any regulatory action to reduce emissions if health risks are confirmed in the future, as is now evident among legacy compounds.^17^

Limited toxicological data are available for replacement PFASs especially for apex predators and humans, but studies on rats, mice, and fish have started to demonstrate that a number of compounds have the potential to cause toxic effects.^22,30,81,82^ Further research is needed on toxicity as well as their potential to bioaccumulate and biomagnify, and thus, the risk to apex predators and humans. As chemical companies continue to innovate, industry confidentiality and the time needed to develop analytical methods mean research and regulatory risk assessment inevitably lag behind production.^83,84^ One of the biggest challenges in targeted PFAS analysis is the lack of reference standards,^7^ and this risks regrettable substitutions.^22,30^ Increased collaboration between industry, research, and regulation is urgently needed.

## Supporting Information Available

Methods: Otter sample selection breakdown (Table S1); further details of analytical determination; list of determinands and analytical standards (Table S2); variables relevant to testing association of PFAS concentrations in otters to distance from PTFE manufacturing site (Table S3); principal component analysis (PCA) results for PFASs which were detected in ≥70% of otters (Table S4); principal component analysis (PCA) biplot for PC1 and PC2 (Figure S1). Results: GLM results for PFASs modeled (Table S5); model predicted decline in PFOA concentration with distance from the factory producing PTFE for the previous data set (2007–2009, blue) and this study’s data set (2015–2019, red) (Figure S2); model predicted decline in PFOA concentration with distance from the factory producing PTFE, without the 3 most southerly located otters (Figure S3); model predicted change in PC1 (largely representative of long chain PFASs which loaded negatively) and PC2 (largely representative of short chain PFASs which loaded positively, and PFOA, L-PFOS and PFOSA which loaded negatively) with distance from the factory and year (Figure S4); principal component analysis (PCA) plots with sex (top) and age (bottom) color coded and ellipses shown (Figure S5)(PDF)
