# Supplementary material for: Persistence of PFOA Pollution at a PTFE Production Site and Occurrence of Replacement PFASs in English Freshwaters Revealed by Sentinel Species, the Eurasian Otter (Lutra lutra)
Source: Environ Sci Technol. 2024 May 27;58(23):10195–206. doi: 10.1021/acs.est.3c09405 (PMC11171452; doi:10.1021/acs.est.3c09405)
Supplement: Supplementary file 1 — es3c09405_si_001.pdf [file es3c09405_si_001.pdf]

Supporting information

Persistence of PFOA pollution at a PTFE production site, and occurrence of replacement PFASs in English freshwaters revealed by sentinel species, the Eurasian otter (*Lutra lutra*)

Emily O'Rourke[a], Sara Losada[b], Jonathan L Barber[b], Graham Scholey[c], Isobel Bain[c], M Glória Pereira[d], Frank Hailer[a] and Elizabeth A Chadwick[a*]

[a] Cardiff University, School of Biosciences, Museum Avenue, Cardiff, CF10 3AX, UK

[b] Centre for Environment, Fisheries and Aquaculture Science (Cefas), Lowestoft, Suffolk, NR33 0HT, UK

[c] Environment Agency, Red Kite House, Howbery Park, Wallingford, Oxfordshire, OX10 8BD, UK

[d] UK Centre for Ecology and Hydrology, Lancaster Environment Centre, Library Avenue, Bailrigg, Lancaster LA1 4AP, UK

*Corresponding author: ChadwickEA@cardiff.ac.uk

12 pages, 5 tables, 5 figures and 1 paragraph (outlining analytical methods)

Methods

- Table S1: Otter sample selection breakdown
- Further details on analytical determination
- Table S2: List of determinands and analytical standards
- Table S3: Variables relevant to testing association of PFAS concentrations in otters to distance from PTFE manufacturing site.
- Table S4: Principal Component Analysis (PCA) results for PFASs which were detected in ≥70% of otters.
- Figure S1: Principal component analysis (PCA) biplot for PC1 and PC2

Results

- Table S5: GLM results for PFASs modelled.

- Figure S2: Model predicted decline in PFOA concentration with distance from the factory producing PTFE for the previous data set (2007-2009, blue) and this study's data set (2015-2019, red).
- Figure S3: Model predicted decline in PFOA concentration with distance from the factory producing PTFE, without the 3 most southerly located otters.
- Figure S4: Model predicted change in PC1 (largely representative of long chain PFASs which loaded negatively) and PC2 (largely representative of short chain PFASs which loaded positively, and PFOA, L-PFOS and PFOSA which loaded negatively) with distance from the factory and year.
- Figure S5: Principal component analysis (PCA) plots with sex (top) and age (bottom) colour coded and ellipses shown.

Methods

Table S1: Otter sample selection breakdown

Variable	Detail of data collection	n / range
Sex	Determined at post-mortem examination (PM) by Cardiff University Otter Project (CUOP).	Male n = 9<br>Female n = 11
Age	Determined at PM by CUOP using following categories:<br>- Female sub-adult = weight ≥2.1kg, immature uterus, no sign of previous/ current reproduction.<br>- Female adult = mature uterus and/or sign of previous/ current reproduction.<br>- Male sub-adult = weight ≥3kg, baculum <60mm.<br>- Male adult = baculum ≥ 60mm.	Sub-adult n = 8<br>Male n = 6<br>Female n = 2<br>Adult n = 12<br>Male n = 3<br>Female n = 9
Body length (mm)	Nose to tail tip. Measured at PM by CUOP.	Range: 885-1180 mm<br>Normal distribution
Body condition	Scaled Mass Index (SMI) estimate of body condition (using Peig and Green, 2009). Calculated from length and weight measurements taken during PM by CUOP.	Range: 5.307-8.771<br>Normal distribution

Further details on analytical determination

Before extraction samples were thawed and homogenised. 1g samples were spiked with 20 µL of a mixture of isotopically mass-labelled recovery/internal standards (ISTDs) in methanol containing 0.2 ng/µL of each ISTD (13C4-PFBuA, 13C5-PFPeA, 13C5-PFHxA, 13C4-PFHpA, 13C8-PFOA, 13C9-PFNA, 13C6-PFDA, 13C7-PFUnDA, 13C2-PFDoDA, 13C2-PFTeDA, 13C8-PFOSA, 13C3-PFBuS, 13C3-PFHxS, 13C8-PFOS, 13C3-HPFOA-DA, d3-NMeFOSAA, d5-NEtFOSAA, 13C2-4:2FTS, 13C2-6:2FTS and 13C2-8:2FTS all from Wellington, Guelph, Canada) in polypropylene tubes. The samples were extracted twice with 5 mL of acetonitrile in an ultrasonic bath (15 min, room temperature). Concentrated extracts underwent dispersive clean-up on 25 mg graphitized carbon (Supelclean ENVI-Carb 120/400, Supelco, Sigma-Aldrich, Stockholm, Sweden) and 50 µL glacial acetic acid in Eppendorf tubes. Aliquots of 0.5 mL of the cleaned-up extracts were diluted with 0.5 mL of 4 mM aqueous ammonium acetate and kept at 4°C until the day of analysis. A 50 times dilution of each sample was also prepared by taking a 0.010 mL aliquot of the cleaned-up extracts and combining it with 0.490 mL of acetonitrile and 0.5 mL of 4 mM aqueous ammonium acetate. The extracts were allowed to warm to room temperature, vortex mixed and centrifuged before the clear solution was transferred to an autoinjector vial, together with 10 µl of a mixture of isotopically mass-labelled injection standards containing 500 ng/µl of 13C4-PFOA and 13C4-PFOS. The analysis of PFAS was performed using an ultra-performance liquid chromatograph Acquity (Waters Ltd, Elstree, Hertfordshire, UK) with an isolator column XBridge C18 (50 mm x 2.1 mm and 3.5 µm particle size), separation was achieved using a BEH C18 analytical column (50 mm x 2.1 mm and 1.7 µm particle size), both columns were from Waters Ltd. The UPLC system was coupled to a TQ MS Xevo triple quadrupole mass spectrometer (Waters Ltd), using an electro spray ionization (ESI) probe in negative mode.

For quality assurance purposes, a blank and reference material sample (NIST 1946 [Lake Superior fish tissue] and NMCAG-RM1 spiked mussel tissue) were analysed with every 10 samples. The blank was a method (lab) blank. A chemical interference in the quantitative ion channel has been detected for PFBA in human placenta samples [1]. The samples in the present study were spiked with labelled PFBA and concentrations recovery corrected, therefore any interference should be affecting native and labelled at the same level, so final concentrations are therefore corrected for any losses or interferences. The same has been carried out for PFPeA and PFHxA. Validations in biota samples, reference materials and interlabs have not shown problems with any of these chemicals, nor problems with recoveries of the labelled chemicals. This could suggest problems may be sample related more than uniquely method or chemical related.

Table S2: List of determinands and analytical standards

Details include the PFAS name, CAS number, abbreviation, carbon number (C_n), whether the PFAS is a legacy (L) or replacement (R) compound, analytical standard used, and limit of quantification (LOQ) measured in µg/kg wet weight achieved during this study. Nomenclature follows that of Buck et al., 2011. Compounds denoted with a * are new to the suite PFASs analysed by Cefas, the results for these compounds can be considered quantitative as they are from a fully calibrated internal standard method, however, caution should be taken as they have not been subject to a full method validation like the original suite.

PFAS name	CAS number	Abbreviation	C_n	Legacy or Replacement	Analytical Standard	LOQ
Perfluoroalkyl carboxylic acids (PFCAs)						
Perfluorobutanoic acid	375-22-4	PFBA	4	R	13C4-PFBuA	0.05
Perfluoropentanoic acid	2706-90-3	PFPeA	5	R	13C5-PFPeA	0.05
Perfluorohexanoic acid	307-24-4	PFHxA	6	R	13C5-PFHxA	0.1
Perfluoroheptanoic acid	375-85-9	PFHpA	7	R	13C4-PFHpA	0.1
Perfluorooctanoic acid	335-67-1	PFOA	8	L	13C8-PFOA	0.05
Perfluorononanoic acid	375-95-1	PFNA	9	L	13C9-PFNA	0.05
Perfluorodecanoic acid	335-76-2	PFDA	10	L	13C6-PFDA	0.1
Perfluoroundecanoic acid	2058-94-8	PFUnA	11	L	13C7-PFUnDA	0.1
Perfluorododecanoic acid	307-55-1	PFDoDA	12	L	13C2-PFDoDA	0.1
Perfluorotridecanoic acid	72629-94-8	PFTrDA	13	L	13C2-PFDoDA	0.1
Perfluorotetradecanoic acid	376-06-7	PFTeDA	14	L	13C2-PFTeDA	0.1
Perfluoroalkyl sulfonic acids (PFSAs)						
Perfluorobutane sulfonic acid	375-73-5	PFBS	4	R	13C3-PFBuS	0.04
Perfluoropentane sulfonic acid *	2706-91-4	PFPeS	5	R	13C3-PFBuS	0.09
Linear Perfluorohexane sulfonic acid	355-46-4	L-PFHxS	6	L	13C3-PFHxS	0.04
Branched Perfluorohexane sulfonic acid *	355-46-4	B-PFHxS	6	L	13C3-PFHxS	0.02
Perfluoroheptane sulfonic acid *	375-92-8	PFHpS	7	L	13C3-PFHxS	0.1

Linear Perfluorooctane sulfonic acid	1763-23-1	L-PFOS	8	L	13C8-PFOS	0.04
Branched Perfluorooctane sulfonic acid *	1763-23-1	B-PFOS	8	L	13C8-PFOS	0.02
Perfluorononane sulfonic acid *	68259-12-1	PFNS	9	L	13C8-PFOS	0.1
Perfluorodecane sulfonic acid	335-77-3	PFDS	10	L	13C8-PFOS	0.1
Perfluoroalkane sulfonamides (FASAs)						
Perfluorobutane sulfonamide *	30334-69-1	PFBSA	4	R	13C8-PFOSA	0.1
Perfluorohexane sulfonamide *	41997-13-1	PFHxSA	6	L	13C8-PFOSA	0.1
Perfluorooctane sulfonamide	754-91-6	PFOSA	8	L	13C8-PFOSA	0.05
N-Alkyl Perfluoroalkane sulfonamido acetic acids (FASAAs)						
N-ethyl perfluorooctane sulfonamido acetic acid *	2991-50-6	N-EtFOSAA	8	L	d5-NEtFOSAA	0.1
N-methyl perfluorooctanesulfonamidoacetate *	2355-31-9	N-MeFOSAA	8	L	d3-NMeFOSAA	0.05
Fluorotelomer sulfonates (FTSs)						
1H,1H,2H,2H-Perfluorohexanesulfonic acid *	757124-72-4	4:2FTS	6	R	13C2-4:2FTS	0.05
1H,1H,2H,2H-Perfluorooctanesulfonic acid *	27619-97-2	6:2FTS	8	R	13C2-6:2FTS	0.1
1H,1H,2H,2H-Perfluorodecanesulfonic acid *	39108-34-4	8:2FTS	10	R	13C2-8:2FTS	0.05
Cyclic PFAS						
Perfluoroethylcyclohexane sulfonate *	646-83-3	PFECHS	8	R	13C8-PFOS	0.05
Ether-PFASs						
Hexafluoropropylene oxide-dimer acid *	13252-13-6	HPFO-DA (Gen-X)	6	R	13C3-HPFOA-DA	0.1
4,8-dioxa-3H-perfluorononanoic acid *	958445-44-8	NaDONA (ADONA)	6	R	13C8-PFOS	0.09
9-chlorohexadecafluoro-3-oxanone-1-sulfonic acid *	73606-19-6	6:2 Cl-PFESA / 9Cl-PF3ONS (F-53B Major)	8	R	13C8-PFOS	0.05
11-chloroeicosafluoro-3-oxaundecane-1-sulfonic acid *	83329-89-9	8:2 Cl-PFESA / 11Cl-PF3OUdS (F-53B Minor)	10	R	13C8-PFOS	0.05

Table S3: Variables relevant to testing association of PFAS concentrations in otters to distance from PTFE manufacturing site.

In our previous study [2], percentage of arable land in 10km radii of otters was retained (non-significant, variable importance: 0.39) via multi-model inference to explain PFOA concentration, as well as distance to the PTFE manufacturing site and wastewater treatment works load (WWTW) within a 10km radius. Consequently, we initially included both WWTW and arable land in the PFOA model for the current study, but for this, sample model validation steps showed a high collinearity [positive association] between arable land and distance from PTFE manufacturing site based on variance inflation factor, calculated using corvif function in the Car package [3]. Percentage arable land was therefore not included in the starting model for the current analysis.

**Variable**	**Detail**	**Data source**
Factory distance	Linear distance from each otter to PTFE manufacturing site (AGC Chemicals Europe Ltd, Lancashire).	Calculated using ArcGIS join tool to factory location. Location acquired from: Composites UK Hub. Organisation Information for AGC Chemicals Europe. Ltd. Available from: https://compositesuk.co.uk/ hub/agc-chemicals-europe-ltd/
Year of otter death	Sample years 2015-2019. 2015 n=3, 2016 n=5, 2017 n=8, 2019 n=4.	Recorded at time of otter collection.
Latitude (Y coordinate - metres North of UK grid origin)	Control for north/south variation of otters along the transect line.	Recorded at time of otter collection.
Mean wastewater treatment works (WWTW) load	Mean load entering WWTW, measured in Population Equivalent (PE), within 10 km radius around each otter. See O'Rourke et al (2022) for further details on data extraction.	European Environment Agency; Waterbase. UWWTD Urban Wastewater Treatment Directive - Reported Data. Available from: https://www.eea.europa.eu/en/ datahub/datahubitem-view/6244937d- 1c2c-47f5-bdf1-33ca01ff1715
Percentage arable land (note; not included in starting models)	Percentage of arable land within the 10 km radius around each otter. Proxy for the application of sewage sludge on arable land.	UK Centre for Ecology and Hydrology Land Cover Map 2015, 25m raster [4].

Table S4: Principal Component Analysis (PCA) results for PFASs which were detected in ≥70% of otters.

First four principal components (PCs) shown, which explained 81.4% of the variation in the dataset. Calculated in R version 4.1.2 (R Core Team 2021). Important loadings are highlighted (positive = yellow, negative = green). Cut off for important loadings was 0.20412 (sum of the squares of all loadings for each PC divided by number of variables; therefore, variables with a larger loading than this value contribute more than one variable's worth of information to the PC and are regarded as 'important'). PFASs are arranged into classes, carbon number (C_n), whether the PFAS is a legacy (L) or replacement (R) compound are provided. Short chain compounds are highlighted blue. Compounds and class are denoted by their abbreviation, see Table S2 for full compound and class names.

Class	PFAS	C_n	Legacy/ Replacement	PC1 (53.5%)	PC2 (12.3%)	PC3 (9.4%)	PC4 (6.2%)
PFCA	PFBA	4	R	0.162	0.247	0.253	0.134
	PFHpA	7	R	-0.118	0.121	-0.369	0.501
	PFOA	8	L	0.024	-0.337	-0.336	0.433
	PFNA	9	L	-0.022	-0.173	0.408	0.470
	PFDA	10	L	-0.216	-0.033	0.305	0.093
	PFUnA	11	L	-0.219	-0.173	0.069	-0.107
	PFDoDA	12	L	-0.242	-0.120	-0.141	-0.235
	PFTrDA	13	L	-0.223	-0.086	-0.301	-0.075
	PFTeDA	14	L	-0.229	-0.109	-0.236	0.031
PFSA	PFBS	4	R	-0.200	0.296	-0.171	-0.003
	PFPeS	5	R	-0.189	0.266	0.031	0.277
	L-PFHxS	6	L	-0.225	0.265	0.050	0.070
	B-PFHxS	6	L	-0.218	0.310	-0.030	0.120
	PFHpS	7	L	-0.229	0.047	-0.009	0.150
	L-PFOS	8	L	-0.226	-0.228	0.091	0.117
	B-PFOS	8	L	-0.227	-0.041	0.187	0.038
	PFNS	9	L	-0.227	-0.143	0.208	0.077
	PFDS	10	L	-0.236	-0.175	-0.038	-0.172
FASA	PFBSA	4	R	-0.189	0.377	0.038	-0.078
	PFHxSA	6	L	-0.237	0.150	0.009	-0.103
	PFOSA	8	L	-0.236	-0.205	-0.161	-0.117
FTS	8.2FTS	8	R	-0.157	-0.202	0.147	0.095
Cyclic PFAS	PFECHS	8	R	-0.224	0.134	0.044	-0.129
Ether-PFAS	F53B Major	8	R	-0.211	-0.141	0.291	-0.126

Figure S1: Principal component analysis (PCA) biplot for PC1 and PC2

Biplot shows all compounds loading negatively on PC1, except PFOA and PFBA. Shorter chain compounds loading heavily positively (>0.20412) on PC2 with the majority of the other compounds loading negatively on PC2.

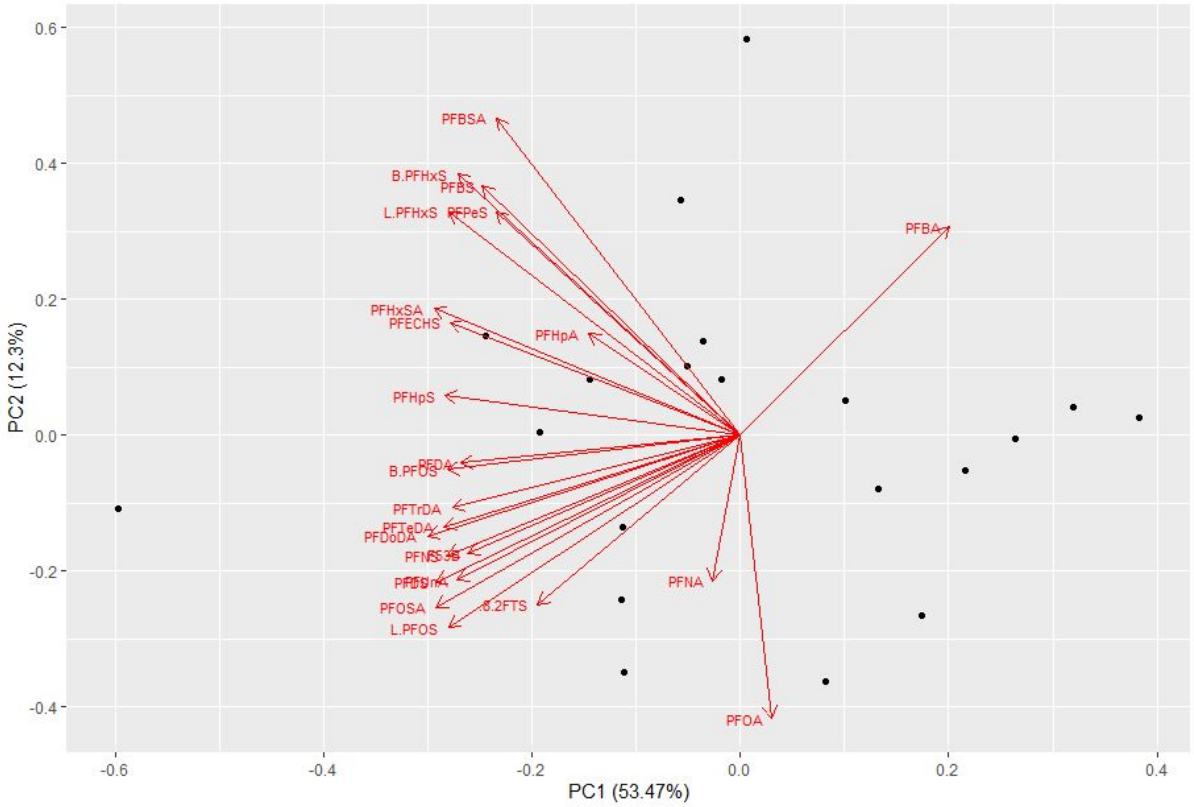

Results

Table S5: GLM results for PFASs modelled.

Significance codes: ‘***’ 0.001, ‘**’ 0.01, ‘*’ 0.05, ‘.’ 0.1, ‘ ’ 1. Standardised estimates presented for averaged model. ‘Estimate’ is the estimated coefficient, and SE is the standard error around that estimate. Models for PFOA and PFNA used raw data with Gamma family and log link function. Models for PFBA and PC1 used raw data with gaussian family and identity link function. All variables in the starting model (factory distance, year, latitude and WWTW load) were included as a continuous variables.

n	Dependent variable (number of top models)	Independent variables retained in top models	Estimate	SE	Adjusted SE	Z value	P value	Significance	N containing models	Relative variable importance
19	PFOA - leverage point removed (2 models)	Factory	-1.38606	0.15241	0.16406	8.449	<2e-16	***	2	1
		Year	-0.07982	0.13824	0.14315	0.558	0.577		1	0.37
20	PFOA – including Leverage point (3 models)	Factory	-0.6376	0.4338	0.4453	1.432	0.1522		2	0.8
		WWTW	0.6803	0.2292	0.2471	2.753	0.0059	**	3	1
		Latitude	0.5277	0.445	0.4549	1.16	0.246		2	0.71
16	PFOA – southern otters removed (3 models)	Factory	-0.67957	0.16417	0.17861	3.805	0.000142	***	3	1
		Latitude	0.09831	0.16593	0.17244	0.57	0.568604		1	0.36
		Year	-0.03665	0.09699	0.10121	0.362	0.717292		1	0.2
20	PC1 (7 models)	Latitude	1.26E+00	1.71E+00	1.76E+00	0.714	0.475		3	0.47
		Year	4.78E-01	1.15E+00	1.19E+00	0.4	0.689		2	0.24
		WWTW	-4.03E-01	1.06E+00	1.10E+00	0.365	0.715		2	0.22
		Factory	-1.47E-01	6.79E-01	7.05E-01	0.208	0.835		1	0.09
20	PFNA (2 models)	Year	-0.2484	0.3071	0.3174	0.783	0.434		1	0.55
20	PFBA (2 models)	WWTW	-0.02949	0.06595	0.06866	0.43	0.668		1	0.3

Figure S2: Model predicted decline in PFOA concentration with distance from the factory producing PTFE for the previous data set (2007-2009, blue) and this study's data set (2015-2019, red).

Blue dots show the raw data for the 2007-09 dataset, the solid blue is the model predicted concentrations. Red dots show the raw data for the 2015-19 dataset, the solid red line is the model predicted concentrations and the orange line shows the predicted concentrations forecast beyond the dataset. Dotted lines show standard error. Other variables retained in the top models of the respective datasets are controlled in the model predictions, see Table S5 for this study and O'Rourke et al. 2022 for previous dataset for details. Note; the datasets were not modelled together due to the differing sample selection (a spread of otters from across England and Wales for 2007-09 data, and a transect east from the factory for 2015-19 data – there was insufficient overlap between the datasets to enable robust comparison within the same model).

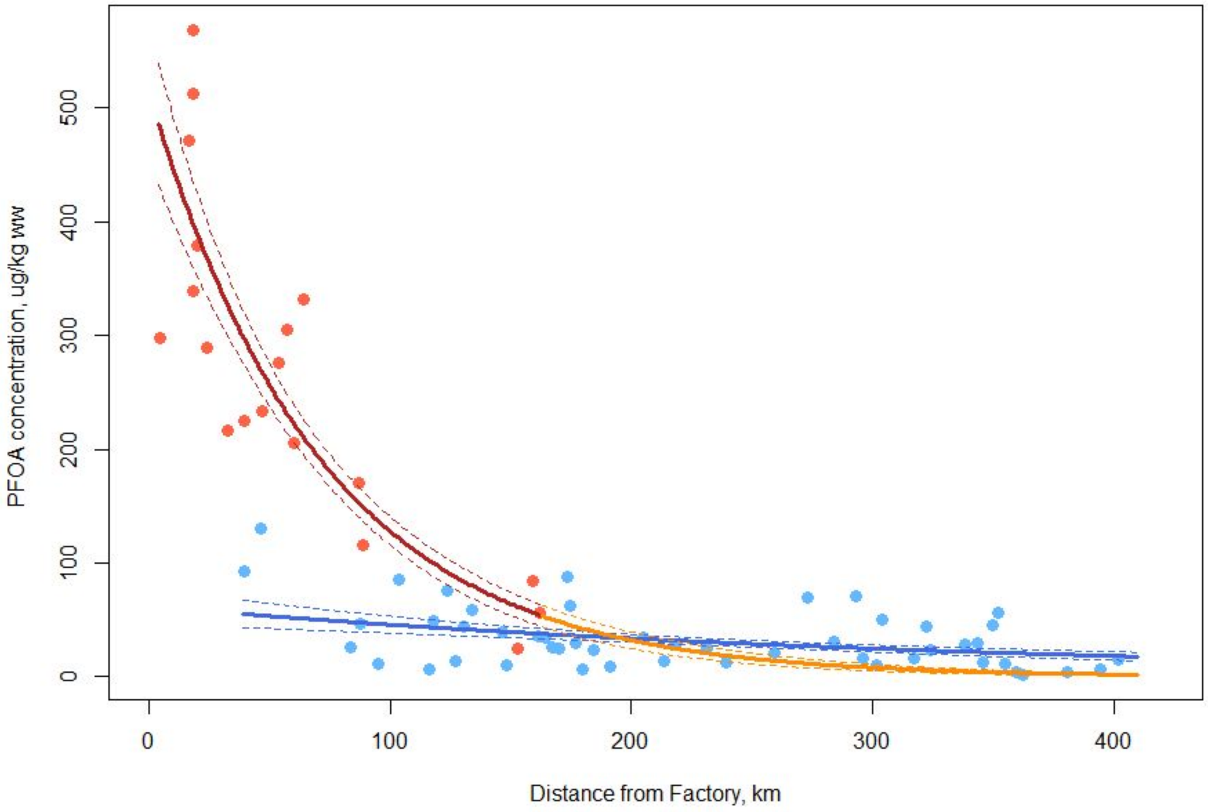

Figure S3: Model predicted decline in PFOA concentration with distance from the factory producing PTFE, modelled with the leverage point (n = 20).

Coloured dots show the raw data, colour-coded by river catchment. Solid red line shows the model predicted concentrations and dotted lines show standard error. Year, latitude and WWTW load are controlled to their mean values, see statistical methods for details. Model results indicate the same trend as our model without the leverage point (n = 19) which is reported in our main text.

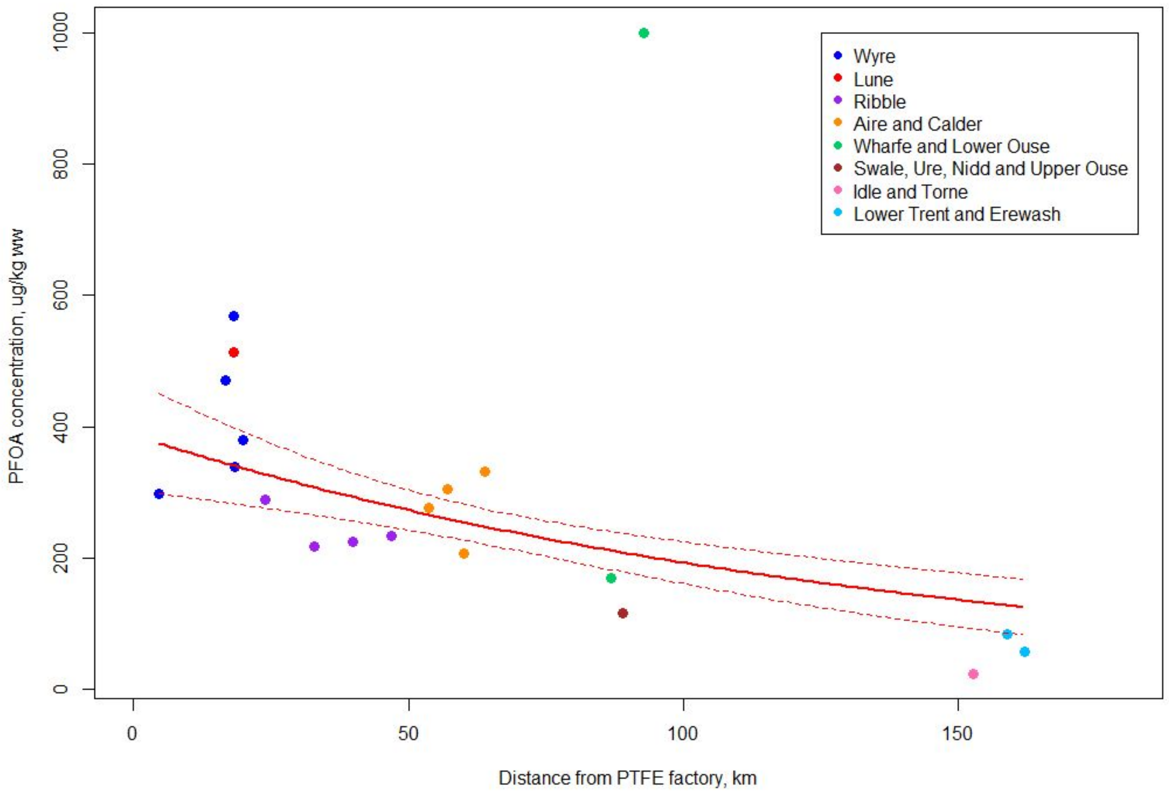

Figure S4: Model predicted decline in PFOA concentration with distance from the factory producing PTFE, without the 3 most southerly located otters (n =16).

Due to the more southerly location of the three most distant individuals along our transect we compared models with and without those individuals to ensure that they did not bias our conclusions. Results presented here are without the 3 most southerly located otters (and without the otter with a high PFOA concentration causing excessive leverage, n = 16). Coloured dots show the raw data, colour-coded by river catchment. Solid red line shows the model predicted concentrations and dotted lines show standard error. Year, latitude and WWTW load are controlled to their mean values, see statistical methods for details.

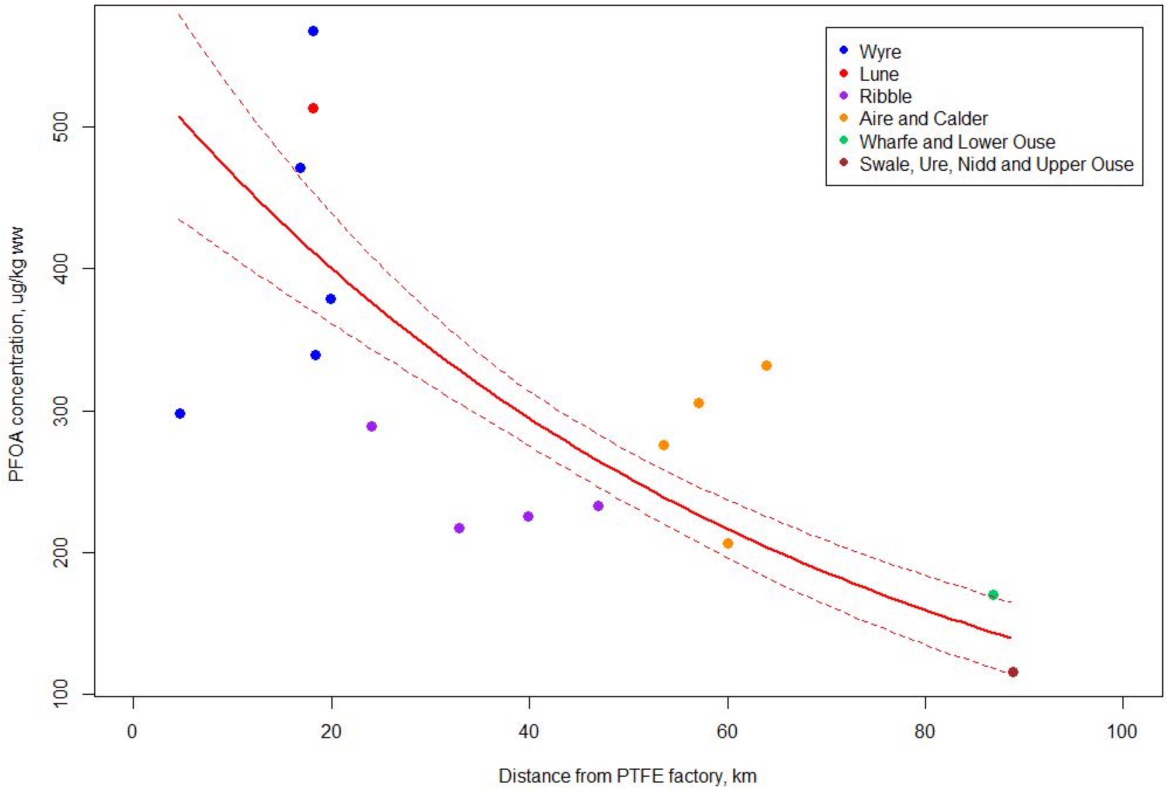

Supporting information references

1. Bangma, J.T., J. Reiner, R.C. Fry, T. Manuck, J. McCord, and M.J. Strynar, Identification of an analytical method interference for perfluorobutanoic acid in biological samples. *Environmental science & technology letters*, 2021. **8** (12): p. 1085-1090.

2. O'Rourke, E., J. Hynes, S. Losada, J.L. Barber, M.G. Pereira, E.F. Kean, F. Hailer, and E.A. Chadwick, Anthropogenic drivers of variation in concentrations of perfluoroalkyl substances in otters (Lutra lutra) from England and Wales. *Environmental Science & Technology*, 2022. **56**(3): p. 1675-1687.

3. Fox, J. and S. Weisberg, An {R} companion to applied regression 3rd ed Sage Thousand Oaks. 2019, CA.

4. Rowlands, C.S., R.D. Morton, L. Carrasco, G. McShane, A.W. O'Neil, and C.M. Wood, Land CoverMap 2015 (25m raster, GB), N.E.I.D. Centre, Editor. 2017.
